# Radiosynthesis of 5-[^18^F]Fluoro-1,2,3-triazoles through Aqueous Iodine–[^18^F]Fluorine Exchange Reaction

**DOI:** 10.3390/molecules26185522

**Published:** 2021-09-11

**Authors:** Xiang Zhang, Falguni Basuli, Sameh Abdelwahed, Tadhg Begley, Rolf Swenson

**Affiliations:** 1 Chemistry and Synthesis Center, National Heart, Lung, and Blood Institute, National Institutes of Health, Rockville, MD 20892, USA; bhattacharyyaf@nhlbi.nih.gov (F.B.); rolf.swenson@nih.gov (R.S.); 2 Department of Chemistry, Prairie View A&M University, Prairie View, TX 77446, USA; shabdelwahed@pvamu.edu; 3 Department of Chemistry, Texas A&M University, College Station, TX 77843, USA; begley@chem.tamu.edu

**Keywords:** 1,2,3-triazole, radiofluorination, positron emission tomography, halogen exchange, thiamin, azeotropic drying

## Abstract

In this report, a simple and efficient process to achieve fluorine-18-labeled 1,2,3-triazole is reported. The heteroaromatic radiofluorination was successfully achieved through an iodine–fluorine-18 exchange in an aqueous medium requiring only trace amounts of base and no azeotropic drying of fluorine-18. This methodology was optimized on a model reaction and further validated on multiple 1,2,3-triazole substrates with 18–60% radiochemical conversions. Using this strategy—the radiosynthesis of a triazole-based thiamin analogue—a potential positron emission tomography (PET) probe for imaging thiamin-dependent enzymes was synthesized with 10–16% isolated radiochemical yield (RCY) in 40 min (uncorrected, *n* > 5).

## 1. Introduction

1,2,3-Triazoles are fundamental building blocks in many bioactive compounds. Generally, triazoles are stable under acidic or basic conditions, metabolic degradation, and redox conditions. They can form H-bonds and π-π stacking interactions that provide diverse pharmacological properties [1]. The unique structural features also enable them to mimic a variety of functional groups such as amides and esters, heterocycles, olefin-rigid analogues, etc. [2]. Therefore, this structure has drawn significant attention in medicinal chemistry [3]. Numerous 1,2,3-triazole bearing structures are reported to have active antimicrobial [4,5,6], antiviral [7,8,9], antioxidant [10,11], and antitumor effects [12,13,14,15], making them important scaffolds in drug development.

In the positron emission tomography (PET) imaging field, this framework has also been extensively utilized as a versatile linker for attaching short-lived radioisotopes. The heterocycle is normally stable under in vivo conditions [16] and also introduces some degree of polarity into the imaging tracer [17]. Additionally, it can be designed as a surrogate for the amide bond [18]. Among the methods of introducing fluorine-18 to a triazole, copper-catalyzed azide-alkyne 1,3-dipolar cycloaddition (CuAAC, click chemistry) is undoubtedly the most powerful approach [19,20,21,22]. The simplicity and highly efficient nature of CuAAC have enabled numerous applications of this process in the radiosyntheses of both small molecules [23,24,25] and macromolecules [26,27,28,29]. However, this method requires multi-step radiosynthesis: the azeotropic drying of fluorine-18, the incorporation of fluorine-18 on the alkyl- or azide- substrates, and the purification of the labeled substrate followed by click reaction with biomolecules. Late-stage, direct fluorination to the triazole ring remains challenging. To our knowledge, no methodology of appending fluorine-18 directly to 1,2,3-triazole heterocycles has been reported.

**Scheme 1:**
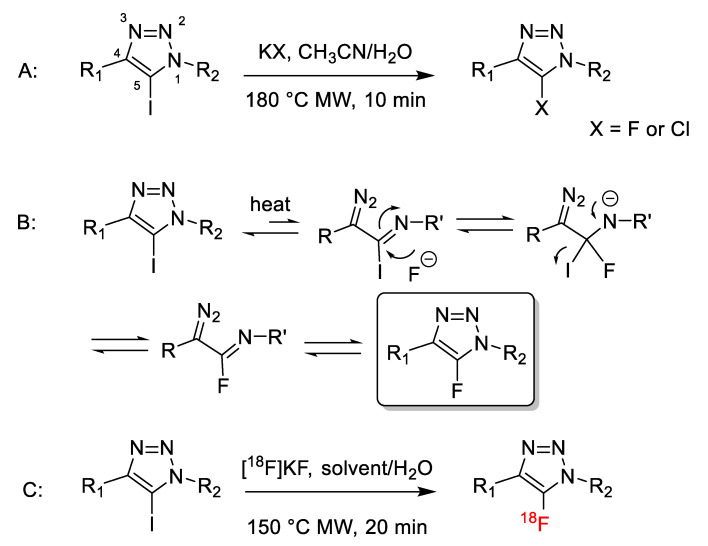
Aqueous Halex reaction of 5-iodo-1,2,3-triazole to give 5-fluoro/chloro-1,2,3-triazole (**A**, literature) [30], the proposed mechanism (**B**, literature) [30] and aqueous [^18^F]IFX approach to synthesize 5-[^18^F]fluoro-1,2,3-triazole (**C**, this work).

In 2012, Fokin et al. first discovered an efficient and straightforward halogen exchange (Halex) reaction of 5-iodotriazoles to prepare 5-fluoro/chloro-triazoles [30]. Under Fokin et al.’s conditions, various fluoro- and chloro-substituted 1,2,3-triazoles were obtained in aqueous media containing a large excess of KF or KCl (Scheme 1A). A ring-opening mechanism was proposed to generate a reactive diazo/imidoyl iodide intermediate which further reacts with fluoride ions (Scheme 1B). Mild fluorinating agents such as KF or KHF_2_, fast reaction times (10 min), aqueous reaction conditions, and excellent functional-group tolerance further enhanced the feasibility of this methodology. Later on, Chu et al. reported the silver-mediated fluorination of 5-iodotriazoles with AgF to prepare 5-fluorotriazoles [31]. Although reaction temperature was lowered (120 °C vs. 180 °C) when using AgF as a source of fluorine, the reaction time became much longer (20 h vs. 10 min). Nevertheless, these aqueous iodine–fluorine exchange (IFX) reactions—which require no time-consuming azeotropic drying of fluorine-18 or phase-transfer reagents—would be beneficial for the production of fluorine-18-labeled PET tracers. In this work, we report the application of this highly efficient heteroaromatic substitution in the radiosynthesis of fluorine-18-labeled 1,2,3-triazole analogs (Scheme 1C), as well as a triazole-based thiamin analogue, a potential PET probe for thiamin-dependent enzyme imaging.

## 2. Results and Discussion

A commercially available compound, 5-iodo-1,4-dimethyl-1*H*-1,2,3-triazole (**1**), was used to optimize the [^18^F]IFX reaction. The [^18^F]IFX radiofluorination was performed by adding co-solvent and [^18^F]HF (in target water) to a microwave vessel containing the precursor **1**. The effects of several factors on radiochemical conversions, such as base, co-solvent, temperature, and reaction time, were investigated (Table 1). First, the use of CH_3_CN was tested. However, due to the high temperature required vs. the low boiling point of CH_3_CN, the pressure in reaction vials can reach the limit of the microwave (MW) reactor and trigger safety cooling. On the other hand, the use of DMF or DMSO increases the radiochemical conversion (RCC, up to 50%, entry 3–10). As a result, we decided to use DMSO as the co-solvent of choice. Various reaction temperatures were also tested without any significant differences in RCCs between 140–160 °C. When the temperature was 130 °C or below, little to no product was observed. This finding is in agreement with the literature suggesting that a minimum of 140 °C is required to initiate the reaction [30].

**Figure 1:**
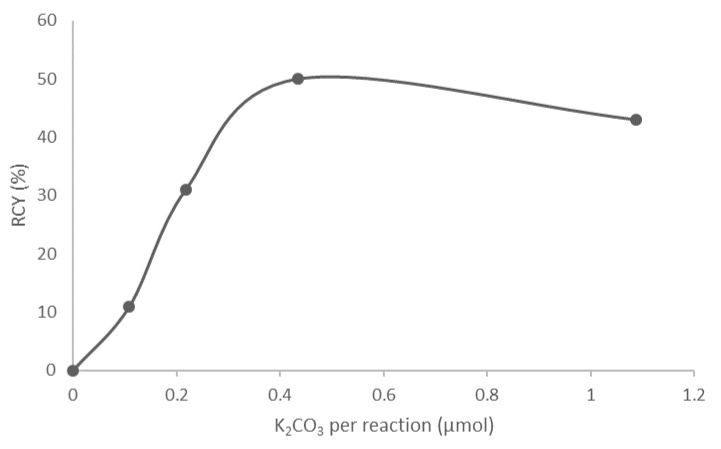
Effect of a variable amount of K_2_CO_3_ on RCC.

The effect of the amount of base was also investigated (entries 3, 5, 6, 7, 9). The reaction did not proceed in the absence of a base. This was expected since [^18^F]HF is known to be non-nucleophilic without neutralization. Since the cyclotron-produced [^18^F]HF had a very low mass concentration, the radiosyntheses were tested with trace levels of K_2_CO_3_. When the reaction was spiked with 15 µg of K_2_CO_3_ (0.11 µmol), 11% RCC was observed. The RCC (31%) was improved by increasing the amount of K_2_CO_3_ to 30 µg (0.22 µmol), and plateaued (50%) at 60 µg/reaction (0.43 µmol, Figure 1). Based on the model reaction, we settled on the following conditions: K_2_CO_3_ (60 µg, 0.43 µmol), DMSO/H_2_O, and 150 °C MW for 20 min.

**Figure 2:**
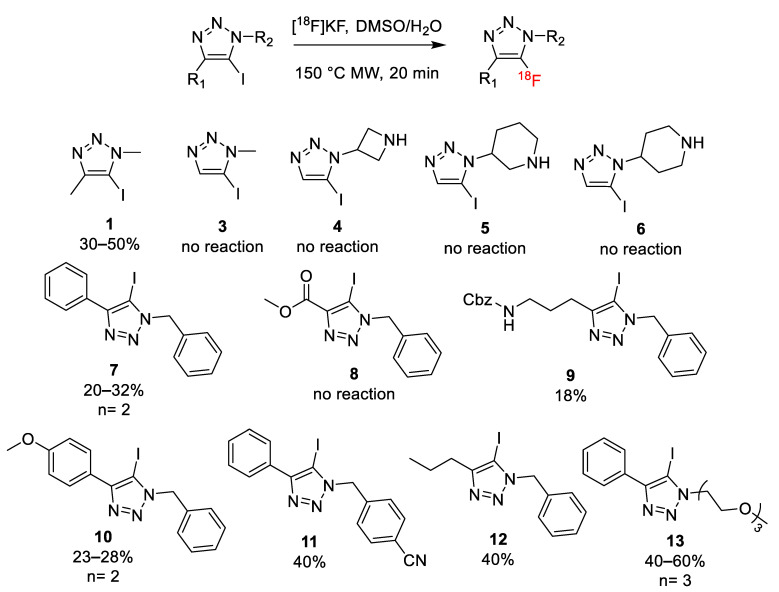
Substrate scope study. Reaction condition: precursor (5 mg), [^18^F]HF in target water (370–740 MBq), K_2_CO_3_ (60 µg, 0.43 µmol), DMSO (0.3 mL), 150 °C MW, 20 min. RCC was determined by analytical HPLC and the identity of the product was confirmed by co-injection with the standard compounds. HPLC condition: Phenomenex Luna C18 (2) column, 100 × 4.6 mm, 5 µm. Mobile phase: 5–95% acetonitrile in water (0.1% NH_4_OH) in 8 min; 1.0 mL/min.

With optimized conditions, a substrate scope study was performed to assess the feasibility of this methodology (Figure 2). For the four substrates without C4 substitution (compound **3**–**6**), no product was observed. This demonstrated that a C4 substitution is necessary, perhaps to stabilize the open diazo form of the intermediate (Scheme 1B) [30]. A variety of 1,4-disubstituted-5-iodo-1*H*-1,2,3-triazoles were designed to further evaluate the application scope (Compounds **7**–**13**). These substrates were readily prepared by following a published procedure [32]. Under the standard condition, most of the radiofluorinated product was obtained in medium to good RCCs. For N1 and C4 substitution, both aromatic and aliphatic groups were well tolerated to produce the desired product. More specifically, for the N1 position, no significant differences between RCCs were noticed for substrates with electron-withdrawing and electron-donating groups. Similar or slightly improved RCC was achieved when the benzyl group of **7** was replaced with a more electron-withdrawing 4-cyanobenzyl group (**11**), or a more electron-donating polyethylene glycol (PEG) group (**13**). However, for the C4 substitution, the replacement of the phenyl group (**7**) with methyl ester (**8**) completely inhibited radiofluorination. This finding is in agreement with the literature, indicating that no transformation occurs for substrates with an electron-withdrawing group on C4 [30]. In contrast to the previously reported examples, we found that both aromatic and aliphatic C4 substituents were able to successfully activate radiofluorination. All different C4 aliphatic substrates (**9**, **12**) produced the desired product (18–40% RCC).

**Figure 3:**
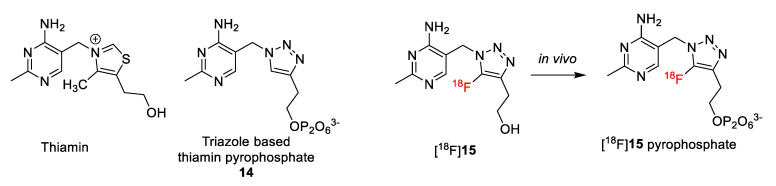
Structure of thiamin, triazole-based thiamin pyrophosphate, and the fluorine-18-labeled triazole based analogue of thiamin undergoing in vivo pyrophosphorylation.

After the success of the aqueous [^18^F]IFX radiofluorination with a wide range of substrates, we focused on the development of a novel PET tracer, [^18^F]**15** (Figure 3), a potential imaging probe for thiamin-dependent enzymes. Thiamin plays a key role in numerous body functions such as energy metabolism, protein and nucleic acid biosynthesis [33]. It is particularly important in the function of the nervous system and protection against neurological disorders [34]. Recent studies have demonstrated the significance of thiamin-dependent enzymes in cancer cell metabolism [35]. This suggests that tumor cells will display an elevated uptake of probes targeting thiamin-dependent enzymes, a finding that may be of diagnostic value in the early detection of cancer through PET. So far, a triazole-based thiamin pyrophosphate, **14**, is one of the most potent inhibitors for thiamin-dependent enzymes [36,37]. We reasoned, therefore, that the fluorine-substituted analog of **14**, [^18^F]**15,** would be an excellent candidate as a PET imaging agent (Figure 3).

**Scheme 2:**
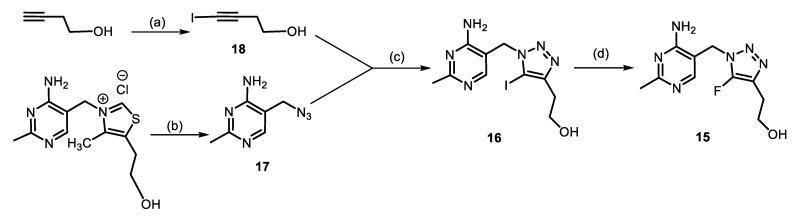
Synthesis of the radiolabeling precursor **18** and non-radioactive standard **15**. Conditions: (**a**) KI, MeOH, TBHP, 2 h; (**b**) NaN_3_, Na_2_SO_3_, H_2_O, 60–65 °C, 6 h; (**c**) CuI, DMF, TEA, 20 h; (**d**) KF, DMSO, 185 °C, 5 min.

The non-radioactive standard compound **15** and the radiolabeling precursor **16** were synthesized as depicted in Scheme 2. The azido intermediate **17** was synthesized from a commercially available thiamin chloride [36]. Iodo-precursor **16** was obtained by the CuAAC reaction of the azido intermediate **17** and the alkynyl compound **18** [38]. Compound **15** was then prepared from the iodo-counterpart by the Halex reaction [30,39].

The [^18^F]IFX was first tested by the traditional radiofluorination approach. Briefly, 5-iodotriazole precursor (**16**) in CH_3_CN or DMSO was reacted with azeotropically dried fluorine-18 ([^18^F]KF/K_2_CO_3_/K_222_ or [^18^F]TBAF/TBAB) at a temperature between 100 and 180 °C. However, no product formation was detected under these conditions. The decomposition of the precursor was observed for all conditions tested, although it is relatively more stable in TBAB vs. K_2_CO_3_/K_222_.

**Scheme 3:**
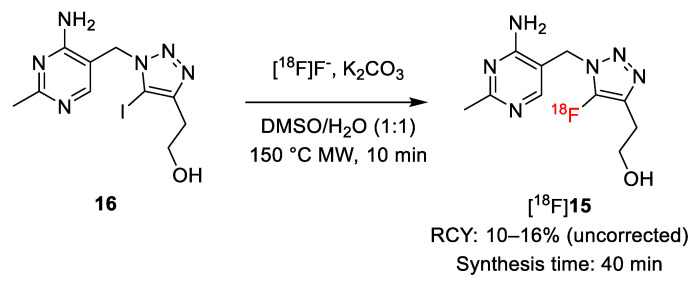
Radiosynthesis of [^18^F]**15**.

**Figure 4:**
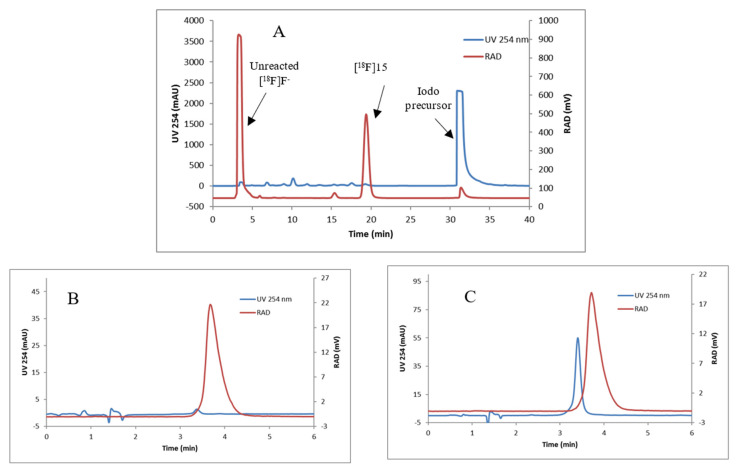
(**A**) Semi-preparative HPLC chromatogram of [^18^F]**15** purification; (**B**) analytical HPLC chromatograms of purified [^18^F]**15**; (**C**) co-injected with the nonradioactive standard. Red line, in-line radiodetector; blue line, UV detector at 254 nm.

Next, we tested the process of aqueous [^18^F]IFX radiofluorination to prepare the compound [^18^F]**15** (Scheme 3). Cyclotron-produced [^18^F]HF in target water was used directly without azeotropic drying. The iodo-precursor (**16**) in DMSO (200 µL) and an aqueous K_2_CO_3_ solution (0.3 mg/mL, 200 µL) were added to a reaction vial containing aqueous [^18^F]HF. The reaction vial was heated at 150 °C for 10 min via microwave irradiation. The crude reaction mixture was evaluated by analytical HPLC, which indicated 30–34% RCC (n = 4). The product was purified by semi-preparative HPLC to produce [^18^F]**15** in 10–16% RCY (uncorrected, *n* > 5) with radiochemical purity >98% (Figure 4A). The synthesis was completed in 40 min, including fluorination and HPLC purification to produce the inject-ready dose. In a typical production starting with 6.03 GBq (163 mCi) of [^18^F]HF, 0.962 GBq (26 mCi) of [^18^F]**15** was received at the end of synthesis. The identity of [^18^F]**15** was confirmed by co-elution with its authentic nonradioactive standard on an analytical HPLC (Figure 4B,C). It is worth noting that [^18^F]IFX was successfully achieved with fully unprotected (amino and hydroxyl groups) iodo-precursor **16**, which further confirmed the functional group compatibility of this radiofluorination method. With the trace amount of fluorine-18 and the strong rates of incorporation, this reaction displayed unusually high chemoselectivity. The functional groups (hydroxyl and amino) and water did not compete with the fluorine-18 as a nucleophile, perhaps suggesting a tight ion pair between [^18^F]F^−^ and the purported C5-N1 imine intermediate, which leads to fluorination.

## 3. Material and Methods

Compounds **3**–**5** were purchased from AstaTech Inc (Bristol, PA, USA). Compounds **7**–**11** were synthesized according to the literature method outlined in [31,32]. All other chemicals and solvents were received from Sigma Aldrich (St. Louis, MO, USA) and used without further purification. Fluorine-18 was received from the National Institutes of Health’s cyclotron facility (Bethesda, MD, USA). Mass spectrometry (MS) was performed on a 6130 Quadrupole LC/MS (Agilent Technologies, Santa Clara, CA, USA), Agilent Technologies instrument equipped with a diode array detector. The ^1^H, ^13^C and ^19^F NMR spectra were recorded on a Varian spectrometer (400 MHz) (Varian, Palo Alto, CA, USA). Chemical shifts (ppm) are reported relative to the solvent residual peaks. High-performance liquid chromatography (HPLC), for purification and analytical analysis, was performed on an Agilent 1200 Series instrument (Agilent Technologies, Santa Clara, CA, USA) equipped with multi-wavelength detectors along with an Eckert and Ziegler B-FC-3500 diode flow count radiodetector (Eckert and Ziegler, Berlin, Germany).

### 3.1. Chemical Synthesis

#### 3.1.1. General Procedure to Synthesize Compounds **12**–**13**

The procedure followed the literature, with minor modifications [32]. Generally, azides (1.65 mmol), terminal alkynes (1.5 mmol), Selectfluor (1.8 mmol), tetraethylammonium iodide (1.65 mmol), DIPEA (1.8 mmol), and CuI (0.15 mmol) were added to H_2_O (8 mL) and stirred for 5–12 h at 30 ℃ (water bath). The reaction was monitored by TLC. After the reaction was completed, the mixture was partitioned between water and ethyl acetate (3 × 10 mL). The organic layer was then combined, dried over Na_2_SO_4_, and the filtrate was concentrated under reduced pressure. The crude products were further purified to produce pure compounds using flash chromatography on silica gel with hexanes/ethyl acetate as the eluent.

##### 1-Benzyl-5-iodo-4-propyl-1*H*-1,2,3-triazole (**12**)

Flash chromatography was performed at gradient elution with hexanes/ethyl acetate, 90:10 to 50:50. Product was obtained as light-yellow solid (120 mg, 24.5% yield); ^1^H NMR (400 MHz, CDCl_3_) δ 7.41–7.32 (m, 3H), 7.32–7.23 (m, 2H), 5.59 (s, 2H), 2.69–2.61 (m, 2H), 1.74 (m, 2H), 0.98 (t, *J* = 7.4 Hz, 3H); ^13^C NMR (101 MHz, CDCl_3_) δ 152.36, 134.57, 128.85, 128.37, 127.72, 78.21, 54.13, 28.09, 22.32, 13.77. MS (ESI) calculated mass for the parent C_12_H_14_IN_3_ 327.17, found 328.10 [M + H]^+^.

##### 5-Iodo-1-(2-(2-(2-methoxyethoxy)ethoxy)ethyl)-4-phenyl-1*H*-1,2,3-triazole (**13**)

Flash chromatography was performed at gradient elution with hexanes/ethyl acetate, 90:10 to 75:25. Product was obtained as off-white solid (220 mg, 35. 3% yield); ^1^H NMR (400 MHz, CDCl_3_) δ 7.98–7.89 (m, 2H), 7.50–7.42 (m, 2H), 7.42–7.35 (m, 1H), 4.63 (t, *J* = 5.9 Hz, 2H), 3.99 (t, *J* = 5.9 Hz, 2H), 3.69–3.54 (m, 6H), 3.54–3.46 (m, 2H), 3.34 (s, 3H); ^13^C NMR (101 MHz, CDCl_3_) δ 149.61, 130.34, 128.52, 127.53, 71.90, 70.79, 70.59, 70.53, 69.25, 59.02, 50.28. MS (ESI) calculated mass for the parent C_15_H_20_IN_3_O_3_ 417.25, found 418.10 [M + H]^+^.

#### 3.1.2. General Procedure to Synthesize Non-Radioactive Standard of Compounds **7**–**13**

The procedure followed the literature with minor modifications [30]. Briefly, the iodide starting material (1 equiv) and KF (5 equiv) were added to a 2–5 mL microwave vial equipped with a magnetic stir bar. To the solid mixture, CH_3_CN and water (1 mL each) were added. The vial was capped with a crimp cap with Teflon septum and this was placed into the microwave reactor at 180 °C for 30 min. After the vial had cooled to room temperature, the crude mixture was diluted with ethyl acetate and water (3 mL each) and extracted with a Pasture pipette. The resulting aqueous phase was extracted two additional times and the combined organic phase was pushed through a Pasture pipette filled with ~3 g of Na_2_SO_4_. Volatiles were removed under reduced pressure and the residue was purified by flash column chromatography on silica gel, with hexanes/ethyl acetate as the eluent, to afford the fluorinated standard compounds.

##### 1-Benzyl-5-fluoro-4-phenyl-1*H*-1,2,3-triazole (non-radioactive standard compound of **7**)

Compound **7** (51 mg, 0.141 mmol) was used as the starting material. Flash chromatography was performed at gradient elution with hexanes/ethyl acetate, 92:8 to 67:33. Product was obtained as light-yellow oil (25 mg, 34.3% yield); ^1^H NMR (400 MHz, CDCl_3_) δ 7.85 (d, *J* = 7.4 Hz, 2H), 7.50–7.31 (m, 8H), 5.50 (s, 2H); ^13^C NMR (101 MHz, CDCl_3_) δ 149.77 (d, *J* = 283.2 Hz), 133.72, 129.27, 129.02, 128.99, 128.75 (d, *J* = 5.0 Hz), 128.29, 128.09, 127.31 (d, *J* = 6.8 Hz), 125.41 (d, *J* = 3.4 Hz), 51.32; ^19^F NMR (376 MHz, CDCl_3_) δ -151.95. MS (ESI) calculated mass for the parent C_15_H_12_FN_3_ 253.28, found 254.10 [M + H]^+^.

##### Benzyl (3-(1-benzyl-5-fluoro-1*H*-1,2,3-triazol-4-yl)propyl)carbamate (non-radioactive standard compound of **9**)

Compound **9** (25 mg, 0.053 mmol) was used as the starting material. Flash chromatography was performed at gradient elution with hexanes/ethyl acetate, 75:25 to 50:50. Product was obtained as light-yellow oil (8 mg, 41.3% yield); ^1^H NMR (400 MHz, cdcl_3_) δ 7.40–7.33 (m, 7H), 7.33–7.27 (m, 3H), 5.36 (s, 2H), 5.09 (s, 2H), 3.25 (q, *J* = 6.6 Hz, 2H), 2.66 (t, *J* = 7.5 Hz, 2H), 1.90 (p, *J* = 7.1 Hz, 2H); ^13^C NMR (101 MHz, CDCl_3_) δ 156.39, 136.58, 133.67, 129.06, 128.76, 128.50, 128.07, 127.89, 110.00, 66.63, 50.98, 40.25, 28.34, 20.72 (d, *J* = 3.1 Hz). Note: Two carbon signals on the triazole ring are absent due to low concentration and C-F coupling; ^19^F NMR (376 MHz, CDCl_3_) δ -156.65. MS (ESI) calculated mass for the parent C_20_H_21_FN_4_O_2_ 368.41, found 369.10 [M + H]^+^.

##### 1-Benzyl-5-fluoro-4-(4-methoxyphenyl)-1*H*-1,2,3-triazole (non-radioactive standard compound of **10**)

Compound **10** (50 mg, 0.128 mmol) was used as the starting material. Flash chromatography was performed at gradient elution with hexanes/ethyl acetate, 90:10 to 75:25. Product was obtained as light-yellow oil (28 mg, 77.0% yield); ^1^H NMR (400 MHz, CDCl_3_) δ 7.82–7.72 (m, 2H), 7.45–7.32 (m, 5H), 7.04–6.94 (m, 2H), 5.48 (s, 2H), 3.85 (s, 3H); ^13^C NMR (101 MHz, CDCl_3_) δ 159.48, 149.03 (d, *J* = 282.0 Hz), 133.63, 129.08, 128.80, 127.92, 127.08 (d, *J* = 6.3 Hz), 126.62 (d, *J* = 3.2 Hz), 121.21 (d, *J* = 4.6 Hz), 114.28, 55.29, 51.11; ^19^F NMR (376 MHz, CDCl_3_) δ -153.30. MS (ESI) calculated mass for the parent C_16_H_14_FN_3_O 283.31, found 284.10 [M + H]^+^.

##### 4-((5-Fluoro-4-phenyl-1*H*-1,2,3-triazol-1-yl)methyl)benzonitrile (non-radioactive standard compound of **11**)

Compound **11** (21.2 mg, 0.075 mmol) was used as the starting material. Flash chromatography was performed at gradient elution with hexanes/ethyl acetate, 90:10 to 75:25. Product was obtained as light-yellow oil (13 mg, 85.1% yield); ^1^H NMR (400 MHz, CDCl_3_) δ 7.85 (dd, *J* = 7.5, 2.1 Hz, 2H), 7.72 (dd, *J* = 8.4, 2.4 Hz, 2H), 7.47 (td, *J* = 8.4, 2.4 Hz, 4H), 7.38 (td, *J* = 7.4, 2.3 Hz, 1H), 5.55 (d, *J* = 2.3 Hz, 2H); ^13^C NMR (101 MHz, CDCl_3_) δ 149.54 (d, *J* = 283.4 Hz), 138.44, 132.96, 128.92, 128.52, 128.42, 128.12 (d, *J* = 4.8 Hz), 127.34 (d, *J* = 6.3 Hz), 125.26 (d, *J* = 3.4 Hz), 118.04, 113.07, 50.30; ^19^F NMR (376 MHz, CDCl_3_) δ -152.24. MS (ESI) calculated mass for the parent C_16_H_11_FN_4_ 278.29, found 279.00 [M + H]^+^.

##### 1-Benzyl-5-fluoro-4-propyl-1*H*-1,2,3-triazole (non-radioactive standard compound of **12**)

Compound **12** (32 mg, 0.098 mmol) was used as the starting material. Flash chromatography was performed at gradient elution with hexanes/ethyl acetate, 90:10 to 75:25. Product was obtained as light-yellow oil (10 mg, 46.7% yield); ^1^H NMR (400 MHz, CDCl_3_) δ 7.39–7.32 (m, 3H), 7.30 (m, 2H), 5.37 (s, 2H), 2.58 (t, *J* = 7.6 Hz, 2H), 1.68 (dq, *J* = 14.8, 7.4 Hz, 2H), 0.94 (t, *J* = 7.4 Hz, 3H). ^13^C NMR (101 MHz, CDCl_3_) δ 133.88, 129.01, 128.66, 127.82, 50.87, 25.65 (d, *J* = 3.6 Hz), 21.66, 13.65. Note: Two carbon signals on the triazole ring are absent due to low concentration and C-F coupling; ^19^F NMR (376 MHz, CDCl_3_) δ -157.32. MS (ESI) calculated mass for the parent C_12_H_14_FN_3_ 219.26, found 220.20 [M + H]^+^.

##### 5-Fluoro-1-(2-(2-(2-methoxyethoxy)ethoxy)ethyl)-4-phenyl-1*H*-1,2,3-triazole (non-radioactive standard compound of **13**)

Compound **13** (50.4 mg, 0.121 mmol) was used as the starting material. Flash chromatography was performed at gradient elution with hexanes/ethyl acetate, 90:10 to 50:50. Product was obtained as yellow oil (29 mg, 77.6% yield); ^1^H NMR (400 MHz, CDCl_3_) δ 7.92–7.82 (m, 2H), 7.53–7.42 (m, 2H), 7.42–7.32 (m, 1H), 4.48 (td, *J* = 5.5, 1.0 Hz, 2H), 3.98 (t, *J* = 5.4 Hz, 2H), 3.69–3.54 (m, 6H), 3.53–3.44 (m, 2H), 3.35 (s, 3H); ^13^C NMR (101 MHz, CDCl_3_) δ 150.18 (d, J = 282.4 Hz), 128.83, 128.73 (d, *J* = 4.8 Hz), 128.02, 126.62 (d, *J* = 6.1 Hz), 125.20 (d, *J* = 3.4 Hz), 71.85, 70.79, 70.57, 70.53, 68.70, 58.97, 47.10 (d, *J* = 2.2 Hz); ^19^F NMR (376 MHz, CDCl_3_) δ -151.69. MS (ESI) calculated mass for the parent C_15_H_20_FN_3_O_3_ 309.34, found 310.20 [M + H]^+^.

##### 4-Iodo-but-3-yn-1-ol (**18**)

To a solution of but-3-yn-1-ol (5.0 g, 71.0 mmol) and potassium iodide (13.0 g, 78.5 mmol) in methanol (30 mL), a 70% solution of aqueous *tert*-butyl hydroperoxide (100 mmol) was added, drop-wise, over 50 min and while stirring at room temperature. The reaction mixture was stirred for an additional one hour and quenched with saturated aqueous Na_2_S_2_O_3_. The product was extracted with ethyl acetate and dried over anhydrous MgSO_4_. The solvent was removed under vacuum and the crude product was purified by column chromatography using a hexane/ethyl acetate mixture to give an oily 4-iodo-but-3-yn-1-ol (9.2 g, 65%), ^1^H NMR δ (400 MHz, CDCl_3_): δ = 3.72 (t, *J* = 6.13 Hz, 2H), 2.63 (t, *J* = 6.01 Hz, 2H), 2.23–2.45 (br s, 1H).

##### 5-Azidomethyl-2-methylpyrimidin-4-ylamine (**17**)

Sodium sulfite (0.39 g, 3.0 mmol) was added to a solution of thiamin chloride (10.0 g, 30.2 mmol) and sodium azide (5.0 g, 76.0 mmol) in water (100 mL). The mixture was stirred for 5 h at 65 °C. Citric acid (22.0 g, 100 mmol) was added to adjust pH ≈ 4 and then the aqueous solution was washed with dichloromethane. Potassium carbonate was added to the aqueous phase to pH ≈ 8, upon which some precipitation of the product occurred. The suspension was filtered, and the filtrate was extracted with ethyl acetate and the combined organic layers were washed with brine, dried over MgSO_4_ and evaporated under reduced pressure. The solid residue was pooled with the precipitate and recrystallized from ethyl acetate/hexane to give the azide derivative as fine needles (3.17 g, 65%), m.p. 150–153 °C; ^1^H NMR δ (400 MHz, CDCl_3_): δ 8.10 (s, 1 H), 5.46 (br s, 2 H), 4.21 (s, 2 H), 2.50 (s, 3H); ^13^C NMR δ (100 MHz, DMSO) 166.2, 161.1, 156.0, 107.2, 47.2, 25.1.

##### 2-[1-(4-Amino-2-methyl-pyrimidin-5-ylmethyl)-5-iodo-1*H*-[1,2,3]triazol-4-yl]-ethanol (**18**)

Under an argon atmosphere, 4-iodo-but-3-yn-1-ol (1.2 g, 6.1 mmol), 5-Azidomethyl-2-methylpyrimidin-4-ylamine (1.0 g, 6.1 mmol), triethylamine (975 µL, 7 mmol), DMF (10 mL), and CuI (1.16g, 6.1 mmol) were successively added to a round conical flask. The mixture was stirred vigorously overnight; ethyl acetate (20 mL) was added to the reaction mixture and it was filtered through a pad of Celite. After the removal of the solvent, the crude product was purified by column chromatography using a hexane/ethyl acetate mixture to afford 1.17 g (69%) of pure product as an off-white powder; ^1^H NMR (400 MHz, MeOD) δ 8.00 (s, 1H), 5.51 (s, 2H), 3.81 (t, *J* = 6.7 Hz, 2H), 2.89 (t, *J* = 6.8 Hz, 2H), 2.42 (s, 3H); ^13^C NMR δ (100 MHz, MeOD) δ 161.76, 161.66, 154.89, 154.84, 83.75, 60.41, 48.21, 30.07, 25.62; MS (ESI) calculated mass for the parent C_10_H_13_IN_6_O 360.15, found 361.02 [M + H]^+^.

##### 2-[1-(4-Amino-2-methyl-pyrimidin-5-ylmethyl)-5-Flouro-1*H*-[1,2,3]triazol-4-yl]-ethanol (**15**)

A mixture of iodo-triazole derivative (50 mg, 0.14 mmol) and KF (40 mg, 0.7 mmol) was added to DMSO (250 uL). The reaction mixture was heated up to 180–190 °C for 5 min; DMSO was blown out by air flow. The crude product was purified using flash silica gel column chromatography (gradient, ethyl acetate/hexanes, 1:1) to afford fluoro-triazole derivative (26 mg, 73%); ^1^H NMR (400 MHz, MeOD) δ 8.07 (s, 1H), 5.41 (s, 2H), 3.81 (t, *J* = 6.6 Hz, 2H), 2.84 (t, *J* = 6.5 Hz, 2H), 2.43 (s, 3H); ^13^C NMR δ (100 MHz, MeOD) δ 167.78, 162.17, 155.24, 149.59, 126.12, 107.07, 59.68, 44.40, 26.67, 23.63; ^19^F NMR (376 MHz, MeOD) δ -154.92. MS (ESI) calculated mass for the parent C_10_H_13_FN_6_O 252.25, found 253.12 [M + H]^+^.

### 3.2. Radiosynthesis

#### 3.2.1. General Procedure for the Synthesis of 5-[^18^F]fluoro-1,2,3-triazoles

An aliquot of [^18^F]HF, in target water (370–740 MBq in 50–100 µL), was added to a microwave vial containing the 5-iodo-precursor (2 mg), co-solvent, CH_3_CN, DMSO or DMF (0.2–0.3 mL), and K_2_CO_3_ solution (0.3 mg/mL). The combined volume of water (from target water + base solution + additional water if necessary) was equal to the volume of co-solvent in the reaction vessel. The mixture was heated at elevated temperature for 10–20 min via microwave irradiation. The crude mixture was sampled for HPLC analysis. HPLC condition: Phenomenex Luna C18 (2) column, 100 × 4.6 mm, 5 µm. Mobile phase: 5–95% acetonitrile in water (0.1% NH_4_OH) in 8 min; 1.0 mL/min.

#### 3.2.2. Radiosynthesis of [^18^F]**15**

In a representative production scale synthesis, an aliquot of [^18^F]HF, in target water (6.03 GBq, 163 mCi in 0.15 mL), was mixed with the 5-iodo-precursor (2 mg in 0.3 mL DMSO) and K_2_CO_3_ solution (60 µg, 0.43 µmol in 0.15 mL H_2_O). The mixture was heated at 150 °C for 10 min via microwave irradiation. To the residue HPLC buffer (2 mL) was added and the product was purified by semi-preparative HPLC. Condition: Phenomenex Luna C18 (2) column, 250 × 10 mm, 10 µm. Mobile phase: 8% EtOH in 10 mM Na_2_HPO_4_; flow rate: 4 mL/min; the product was collected between 19 and 20 min. An aliquot of the product was sampled for HPLC analysis. Condition: Phenomenex Luna C18 (2) column, 100 × 4.6 mm, 5 µm. Mobile phase: 5% acetonitrile in water (0.1% NH_4_OH); flow rate: 1 mL/min. The radiochemical yield was 16.0% (uncorrected), the radiochemical purity was >98%, and the molar activity at the end of synthesis was 73.2 GBq/µmol (1.98 Ci/µmol). The synthesis was completed in ~40 min.

## 4. Conclusions

Based on the condition developed by Fokin et al. we have introduced a direct radiofluorination of 1,2,3-triazole using an aqueous [^18^F]IFX reaction. The process is simple, highly efficient, and has significant functional group tolerance. Substrates with amino, hydroxyl groups, or carbamates are tolerated. The chemoselectivity should allow the fluorination of hydrophilic substrates that were not easily radiolabeled previously. The combination of iodo-alkyne/azide click chemistry and aqueous radiofluorination provides a rapid and versatile methodology for developing new PET agents. The [^18^F]IFX reaction was optimized by a model reaction followed by validation on multiple 1,2,3-triazole substrates. Utilizing this new method, a triazole-based thiamin analogue [^18^F]**15**, a potential PET probe for imaging thiamin-dependent enzymes, was successfully synthesized in 40 min with 10–16% RCY. We believe this methodology for late-stage, direct radiofluorination of 1,2,3-triazoles is a significant addition to the CuAAC.

## Figures and Tables

**Table 1:** The model reaction of aqueous [^18^F]IFX radiofluorination *^a^*.

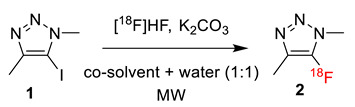					
Entry	Base	Co-solvent	T (MW, °C)	Time (min)	RCC *^b^* (%)
1	K_2_CO_3_	CH_3_CN	150	10	22
2	K_2_CO_3_	CH_3_CN	160	10	40
3	K_2_CO_3_	DMSO	150	20	31
4	K_2_CO_3_	DMSO	160	20	20
5	No base	DMSO	150	20	n/a
6	K_2_CO_3_ *^c^*	DMSO	150	20	11
7	K_2_CO_3_ *^d^*	DMSO	150	20	50
8	K_2_CO_3_ *^d^*	DMSO	140	20	50
9	K_2_CO_3_ *^e^*	DMSO	150	20	43
10	K_2_CO_3_	DMF	150	20	43

Condition: *^a^* precursor (5 mg), K_2_CO_3_ (30 µg, 0.22 µmol) unless otherwise noted, 0.37–0.76 GBq of [^18^F]fluoride in target water (0.2–0.3 mL) and co-solvent (0.2–0.3 mL) were used for each reaction. *^b^* RCCs were determined by analytical HPLC. HPLC condition: Phenomenex Luna C18 (2) column, 100 × 4.6 mm, 5 µm. Mobile phase: 5–95% acetonitrile in water (0.1% NH_4_OH) in 8 min; 1.0 mL/min. *^c^* 15 µg K_2_CO_3_ (0.11 µmol). *^d^* 60 µg K_2_CO_3_ (0.43 µmol). *^e^* 150 µg K_2_CO_3_ (1.09 µmol).

## Data Availability

Not applicable.

## Sample Availability

Samples of the compounds are not available from the authors.
